# Treatment Strategies for Anti-VEGF Resistance in Neovascular Age-Related Macular Degeneration by Targeting Arteriolar Choroidal Neovascularization

**DOI:** 10.3390/biom14030252

**Published:** 2024-02-21

**Authors:** Yingbin Fu, Zhao Zhang, Keith A. Webster, Yannis M. Paulus

**Affiliations:** 1 Cullen Eye Institute, Baylor College of Medicine, Houston, TX 77030, USA; zhaozhang1126@hotmail.com (Z.Z.); kwebster@med.miami.edu (K.A.W.); 2 Department of Neuroscience, Baylor College of Medicine, Houston, TX 77030, USA; 3 Vascular Biology Institute, University of Miami Miller School of Medicine, Miami, FL 33136, USA; 4 Department of Ophthalmology and Visual Sciences, University of Michigan, Ann Arbor, MI 48105, USA; ypaulus@med.umich.edu; 5 Department of Biomedical Engineering, University of Michigan, Ann Arbor, MI 48105, USA

**Keywords:** choroidal neovascularization, CNV, anti-VEGF resistance, neovascular age-related macular degeneration, AMD, arteriolar CNV, anti-VEGF therapies, capillary CNV, AIBP, apoA-I

## Abstract

Despite extensive use of intravitreal anti-vascular endothelial growth factor (anti-VEGF) biologics for over a decade, neovascular age-related macular degeneration (nAMD) or choroidal neovascularization (CNV) continues to be a major cause of irreversible vision loss in developed countries. Many nAMD patients demonstrate persistent disease activity or experience declining responses over time despite anti-VEGF treatment. The underlying mechanisms of anti-VEGF resistance are poorly understood, and no effective treatment strategies are available to date. Here we review evidence from animal models and clinical studies that supports the roles of neovascular remodeling and arteriolar CNV formation in anti-VEGF resistance. Cholesterol dysregulation, inflammation, and ensuing macrophage activation are critically involved in arteriolar CNV formation and anti-VEGF resistance. Combination therapy by neutralizing VEGF and enhancing cholesterol removal from macrophages is a promising strategy to combat anti-VEGF resistance in CNV.

## 1. Limitation of Anti-VEGF Therapies

Age-related macular degeneration (AMD) stands as the leading cause of irreversible blindness among the elderly. In 2020, the number of individuals affected by AMD reached 196 million, with a projection to escalate to 288 million by 2040, imposing a substantial burden on global healthcare systems [1]. Neovascular AMD (nAMD), also known as choroidal neovascularization (CNV), constitutes 10–20% of AMD cases, yet it is responsible for 80–90% of AMD-related blindness [2]. The primary approach in current first-line therapy focuses on inhibiting vascular endothelial growth factor (VEGF), a potent angiogenic factor that stimulates vessel growth and increases vascular permeability.

Despite advancements, up to 50% of patients exhibit incomplete responses to existing anti-VEGF treatments, characterized by persistent fluid, unresolved or new hemorrhage, with suboptimal long-term outcomes even among those who initially respond [3,4,5,6,7,8,9,10,11,12,13,14]. Notably, studies such as Comparisons of Age-Related Macular Degeneration Treatments Trials (CATT) demonstrated persistent retinal fluid accumulation in a substantial percentage of patients treated with bevacizumab (67.4%) and ranibizumab (51.5%) after two years of treatment [3]. In the VIEW 1 and VIEW 2 trials, 19.7–36.6% of patients experienced active exudation after one year of regular 2.0 mg aflibercept treatments [6]. The SEVEN-UP study, focusing on patients exiting the MARINA or ANCHOR trials, revealed a gradual decline in mean visual acuity during long-term follow-up with *pro re nata* (PRN) retreatment [7]. Even patients initially responsive to treatment face challenges, as resistance can develop over time (known as tachyphylaxis) [4,15,16,17]. Studies on nAMD patients treated with bevacizumab showed a gradual decline in response, unaffected by increased dosage [18,19,20]. Similarly, patients treated with ranibizumab experienced recurrence in 66% to 76% of cases following 12–24 months of repeated treatment [21,22].

Various strategies, such as high-dose treatment [19,23,24,25] and switching between anti-VEGF biologics [26,27], have been explored in small-scale studies to address anti-VEGF resistance, demonstrating some success over limited follow-up periods. However, the initial anatomical improvements achieved through the transition to higher-dose therapy tend to plateau over time, resulting in only moderate enhancements in central retinal thickness (CRT) and minimal or negligible gains in visual acuity [25,28]. Studies have shown mild improvement in CRT with either no or only small gains in visual acuity following these interventions [26,28]. In a recent National Institute of Health (NIH)-sponsored trial comparing high doses of bevacizumab, ranibizumab, and aflibercept for treatment-resistant nAMD, no significant benefits were observed in any group, and there was no reduction in injection frequency (remaining at one injection every 5.7–6.4 weeks) [29]. Considering the lack of a substantial response and the potential theoretical risks associated with higher-volume injections, further research is recommended before advocating for the use of even higher dosages of these anti-VEGF agents delivered via standard formulations. Intriguingly, there is a notable similarity in the response to higher dosages of the same therapy and the approach of anti-VEGF switching. This observation suggests that additional common mechanisms contribute to anti-VEGF resistance that are not effectively addressed solely by targeting VEGF.

Combination therapies that simultaneously target VEGF and alternate pro-angiogenic signaling pathways have been explored in clinical trials. Attempts to combine ranibizumab with pegpleranib (Fovista) or nesvacumab, acting as antagonists for platelet-derived growth factor (PDGF) or angiopoietin 2 (Ang2), respectively, failed to meet endpoints [30,31]. Faricimab (Vabysmo), a bispecific antibody targeting both VEGF-A and Ang2, administered at extended treatment intervals (every 16 weeks), demonstrated clinical equivalence (i.e., “no inferiority”) to aflibercept given at 8-week intervals for nAMD, thereby reducing treatment burden for patients [32]. However, there is no evidence showing that faricimab provided significantly improved benefits in treating anti-VEGF-resistant patients. In fact, a recent study has shown that anti-VEGF treatment (e.g., aflibercept, brolucizumab, ranibizumab) also suppresses several key growth factors such as PDGF and Ang2, thus the combined suppression of VEGF and PDGF or Ang2 may not provide optimal clinical benefits [33]. The durability advantage of faricimab may be partly accounted for by the higher molar doses of ranibizumab (the anti-VEGF arm of faricimab is ranibizumab [34]) [33]. Consistent with this, high-dose (8 mg) aflibercept (Eyelea HD) showed similar 12–16-week extended dosing intervals as faricimab in the PULSAR trial. Most ongoing clinical trials continue to target the VEGF pathway without addressing the mechanism(s) causing anti-VEGF resistance. Thus, the development of an effective therapy addressing anti-VEGF resistance remains a critical unmet clinical need.

## 2. Animal Models of Anti-VEGF Resistance

**Figure 1:**
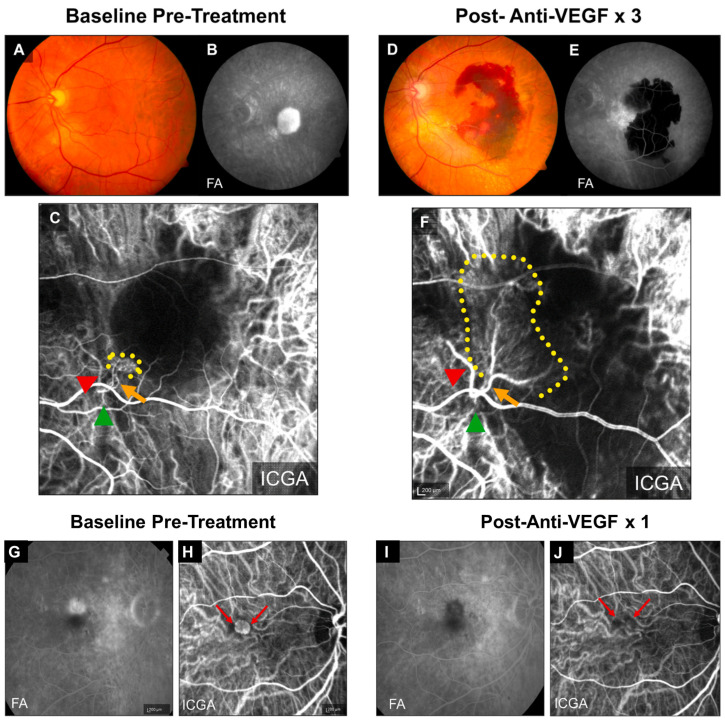
Clinical examples of treatment responses in arteriolar CNV and capillary CNV. (**A**–**F**) Arteriolar CNV. At baseline, (**A**) fundus photography and (**B**) fluorescein angiography (FA) demonstrate evidence of serous pigment epithelial detachment, (**C**) indocyanine green angiography (ICGA) demonstrates an arteriolar predominant lesion, with feeder artery (red arrowhead), arteriole (orange arrow), ill-defined marginal rim of vessels (yellow-dotted region, probable capillaries), and draining vein (green arrowhead). Post-loading dose with three anti-VEGF treatments, (**D**) there is large submacular hemorrhage in the macula by clinical exam and fundus photography, (**E**) FA demonstrates blockage of fluorescence from the hemorrhage but increased late hyperfluorescence at the margin with expanding, blurry margins consistent with leakage from CNV, and (**F**) ICGA demonstrates growth of the CNV lesion, with increased vessel caliber of choroidal feeder artery (red arrowhead), growth of new branching arterioles (orange arrow), extension of arterioles with vascular loops without visible capillaries into the macula (yellow-dotted region), and draining venule (green arrowhead). (**G**–**J**) Capillary CNV. At baseline, (**G**) FA demonstrates a Type 2 CNV pattern and (**H**) ICGA demonstrates capillary CNV morphology (red arrows). Post-treatment with a single anti-VEGF, (**I**) FA shows clearance of the CNV and (**J**) ICGA shows regression of the capillary microvascular structure (red arrows). Used with permission of Elsevier Science and Technology Journals, from Ref. [9]; permission conveyed through Copyright Clearance Center, Inc.

Multiple pivotal clinical trials (ANCHOR, MARINA, CATT) have shown that patients of advanced age with larger baseline CNV lesions are less responsive to anti-VEGF treatment and have worse outcomes [13,35,36,37]. Importantly, anti-VEGF resistance in CNV patients is frequently associated with arteriolar CNV, characterized by large-caliber branching arterioles, vascular loops, and anastomotic connections (Figure 1A–F) [9]. The persistence of fluid leakage in arteriolar CNV likely results from increased exudation through poorly formed tight junctions at arteriovenous anastomotic loops, particularly during periods of elevated blood flow. In contrast, individuals responding well to anti-VEGF treatment typically exhibit capillary CNV, where VEGF-mediated permeability is the primary cause of leakage (Figure 1G–J).

Moreover, recurrent anti-VEGF treatment can induce vessel abnormalization, arteriolar CNV formation, and ultimately contribute to anti-VEGF resistance [14,38], suggesting a mechanism for acquired anti-VEGF resistance.

**Figure 2:**
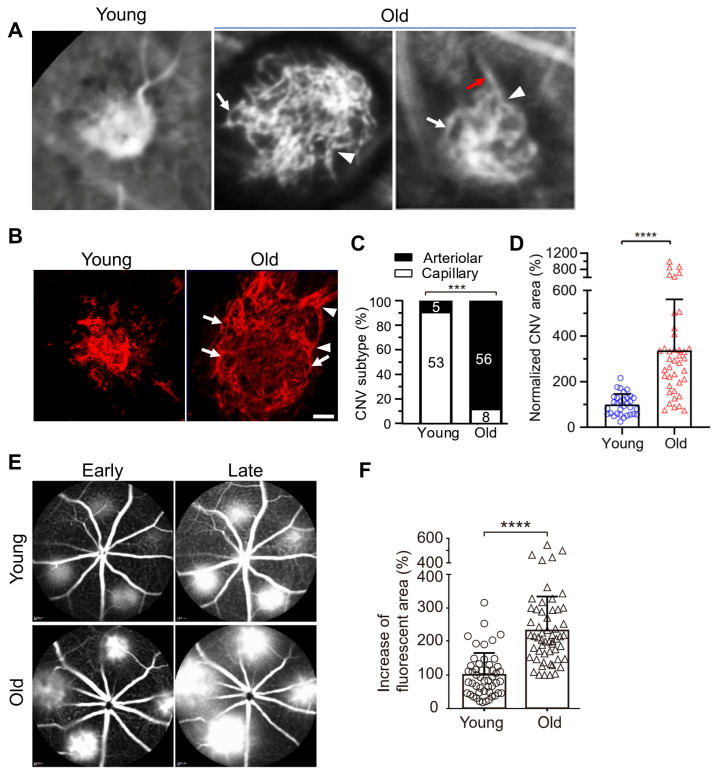
Vascular morphology of laser-induced CNV in young and old mice. (**A**) ICGA of laser-induced CNV in young and old mice. White arrows and arrowheads indicate vascular loops and branching arterioles in old mice, respectively. Red arrows indicate a large caliber feeder vessel. (**B**) Representative images of CNV lesions labeled by Alexa 568 isolectin on RPE/choroid flatmounts in young and old mice. White arrowheads and arrows indicate branching arterioles and vascular loops in old mice, respectively. Scale bar = 40 μm. (**C**) Distribution of capillary and arteriolar CNV in young versus old mice based on ICGA. The numbers inside the bars indicate the number of CNV laser spots. ***, *p* < 0.001. (**D**) Quantitative results of normalized CNV area in young and old mice. CNV areas were measured from Alexa 568 isolectin labeled RPE/choroid flatmounts. *N* = 32 and 40 laser spots in young and old mice, respectively. Bars represent mean ± SD. ****, *p* < 0.0001. (**E**) Early and late phase FA show that laser-induced CNV in old mice exhibits significantly increased hyperpermeability compared with that in young mice. (**F**) The percentage increase of fluorescent area of CNV between the early and late phases of FA. Bars represent mean ± SD. ****, *p* < 0.0001. Adapted from Ref. [40].

We found that laser photocoagulation produces larger CNV lesions in aged mice that are markedly more resistant to anti-VEGF treatment compared with young mice [39,40,41]. Importantly, laser-induced CNV in young and old mice, respectively, mimics capillary and arteriolar CNV (Figure 2) [9,40]. We propose that laser-induced CNV in aged mice is a clinically relevant model of anti-VEGF resistance [39,40]. Although this model uses aging as the pathological driver [40,41], we do not mean to suggest that age is the only factor dictating experimental or clinical CNV or the response to anti-VEGF therapy. In fact, previous studies have shown that cigarette smoking, environmental co-factors (e.g., viral infection), and pathogen-associated molecular patterns (PAMP) stimulation increase the extent and severity of experimental CNV with increased arteriolar CNV formation [42,43,44]. The common underlying theme is macrophage activation, which is consistent with our hypothesis that macrophages play a key role in anti-VEGF resistance (see below) [39,40]. Several main differences exist between mouse models and human AMD patients. Firstly, C57Bl6/J mice have an inbred homogeneous genetic background, whereas human AMD patients carry a wide range of diverse genetic risk factors. It is known that genetic risk factors (e.g., risk alleles in CFH and ARMS2/HTRA1) influence the response to anti-VEGF therapies [45,46,47]. Secondly, laboratory mice inhabit strictly controlled, germ-free environments with a regulated, standardized diet, lighting, etc. This is different from human patients, in which environmental factors, including cigarette smoking and diet, contribute to AMD severity. However, age is also an important risk factor, as multiple pivotal clinical trials have shown that patients of advanced age with larger baseline CNV lesions are less responsive to anti-VEGF treatment and have worse outcomes [13,35,36,37]. This is consistent with our data that laser photocoagulation produces larger CNV lesions in aged mice, and these mice are markedly more resistant to anti-VEGF treatment than young mice [39,40]. Clearly, multiple genetic and environmental factors confound the age effect in human AMD. This explains why aged individuals with nAMD include both responders and non-responders. The important aspect is that laser-induced CNV in aged mice mimics the arteriolar CNV that is resistant to anti-VEGF treatment in human patients, which is invaluable for translational studies. A parallel example is laser-induced CNV, which is the most widely used model of wet AMD (e.g., in rodents, pigs, and nonhuman primates) not only for mechanistic studies but also for most preclinical treatment evaluation experiments. Although laser injury is not a risk factor for AMD, and the model does not have the age-related progressive pathology of nAMD, it captures many of the important features of the human condition, such as newly formed neovascular vessels that project into the subretinal space through defects in Bruch’s membrane and leukocyte infiltration near CNV lesions [48,49,50].

Food and Drug Administration (FDA) guidelines require that the efficacy of a pharmaceutical product be demonstrated in two animal species (one rodent and one non-rodent). While the FDA does not specify species, they require that Investigational New Drug (IND) applicants justify the choice of animal models based on scientific rationale with adequate data to demonstrate the safety and efficacy of the drug candidate in the preclinical models before proceeding to human trials.

Due to their relatively large size, rabbit, canine, porcine, and non-human primate eyes are longstanding retinal models of ocular pathology, including AMD, sharing physiologic parameters comparable with those of humans [51]. These similarities encompass size, internal structure, optical system, biomechanics, and biochemical features. The rabbit eye’s axial length is approximately 80% of that of humans (18.1 mm compared to approximately 23 mm), while the axial lengths of mouse and rat eyes are 3.2 mm and 5.98 mm, respectively, equating to 13% and 24% of humans. Consequently, physiologic manipulation technologies developed for human eyes can be applied to rabbits with minimal modification [52,53,54].

Much is known about the rabbit choroidal vasculature, which shares many similarities with humans [55,56]. Because anti-VEGF resistance models of AMD have not been established in any large mammalian species, we developed a rabbit CNV model of anti-VEGF resistance for drug development. Consistent with our mouse studies, we found that Matrigel and VEGF-induced CNV in aged rabbits are resistant to anti-VEGF treatments (i.e., bevacizumab) [57]. Matrigel-induced CNV in rabbits is considered a closer model of human CNV compared with laser-induced models, in part because Matrigel mimics the sub-RPE drusen deposits that are associated with human CNV [55,58,59,60,61,62].

Considering the larger eye size and longer half-life and residence time of therapeutics, the rabbit is positioned as an appropriate model for efficacy, pharmacokinetics, and toxicology studies. Indeed, studies for ranibizumab, aflibercept, and brolucizumab were performed in rabbits [33,53,63,64,65]. We propose that the two animal models of CNV (i.e., mouse and rabbit) with resistance to anti-VEGF therapies replicate the anti-VEGF resistance of human AMD and constitute valid models to test new therapies. Currently, no other animal models are available for this purpose. Because no animal models fully recapitulate all features of nAMD, successful strategies that have demonstrated promise in alleviating anti-VEGF resistance in aged animals must be evaluated in clinical trials.

## 3. Capillary CNV versus Arteriolar CNV

The formation of capillary CNV shares features with capillary angiogenesis, wherein new capillary blood vessels sprout from preexisting vessels [9,66,67]. Retina ischemia upregulates VEGF (from RPE, Müller cells, infiltrating macrophages, etc.), which binds to the VEGFR2 receptor of endothelia cells to initiate new capillary vessel growth in the subretinal or sub-RPE space [31,68,69].

In contrast, arteriolar CNV formation shares common features with arteriogenesis—the growth and proliferation of pre-existing collateral arteries through remodeling of the vessel wall [70,71]. Unlike angiogenesis, which is highly dependent on VEGF, arteriogenesis is not VEGF-dependent and is mainly driven by shear stress from blood flow [72,73,74]. Arteriogenesis involves endothelial cell activation, basal membrane degradation, leukocyte invasion, proliferation of vascular cells, neointima formation, and changes of the extracellular matrix [75].

Markers of capillary and arteriolar CNV are commonly identified by ICGA and Optical Coherence Tomography Angiography (OCTA), which distinguishes capillary from arteriolar CNV based on the size, shape, and pattern of the lesions. Capillary CNV is characterized by slow-filling, capillary-type microvessels. Arteriolar CNV is characterized by high-flow, large-caliber feeder arteries that give rise to many branching arterioles and anastomotic loop connections with minimal capillary components [9].

Compelling evidence supports functional roles for monocytes and macrophages that infiltrate areas of collateral vessel development and orchestrate arteriogenic remodeling. For example, monocyte depletion in both rabbit and mouse models of hindlimb ischemia leads to impaired arteriogenesis that can be restored by injecting exogenous monocytes [76]. The effect is similar to the depletion of circulating monocytes in murine models that abrogates arteriolar but not capillary CNV in old nAMD mice (see below) [39].

Macrophages play crucial roles in arteriogenesis through multiple pathways, including upregulating matrix metalloproteinases (MMPs) that are essential for collateral artery growth. MMPs are present in and around growing collateral arteries and promote extracellular matrix remodeling and breakdown of the basement membrane. Macrophages also release cytokines such as TNFα that increase leukocyte recruitment and stimulate the proliferation of endothelial and smooth muscle cells by secreting bFGF, PDGF, and VEGF [77].

Based on OCTA studies of CNV patients treated with recurrent anti-VEGF therapies, Spaide proposed a mechanism of arteriolar CNV formation [14]. Anti-VEGF therapy closes many newly formed vessels, increasing vascular resistance for the entire vascular circuit. Increased vessel wall stress induces arteriogenesis in the remaining vessels, leading to an increase in vessel size. When anti-VEGF drugs wane, vascular sprouts regrow. Repeated anti-VEGF treatment prunes back new vessels and reinitiates the cycle. The result is the formation of high-flow, large-caliber vessels, branching arterioles, vascular loops, and anastomotic connections. Thus, arteriolar CNV formation is attributed to the treatment of current anti-VEGF therapies—periodic pruning of angiogenic vascular sprouts by VEGF inhibition with unchecked arteriogenesis [14].

While the precise mechanism for arteriolar CNV formation induced by laser photocoagulation in old mice is still unclear, our results indicate that it involves active vascular remodeling mediated by macrophages, a feature of arteriogenesis in human CNV that is not controlled by VEGF as it is resistant to VEGF neutralization (see “Section 4” below). The mechanistic difference between capillary and arteriolar CNV formation explains why capillary CNV is highly responsive to anti-VEGF treatment, whereas arteriolar CNV is resistant in mouse and rabbit CNV models, as well as human CNV. Despite this, current nAMD drugs only target angiogenesis, with minimal or no effects on arteriogenesis. Therefore, it is important to develop a next generation of therapy that targets both angiogenesis and arteriolar CNV.

## 4. Role of Macrophages in Anti-VEGF Resistance

Several lines of evidence suggest that the accumulation of intracellular lipids in old macrophages plays a critical role in anti-VEGF resistance. Firstly, decreased efficacy of anti-VEGF therapy with age correlates inversely with an age-dependent increase in intracellular lipids in macrophages [39]. Secondly, macrophage depletion in old mice converts arteriolar CNV to capillary CNV [9] and restores CNV sensitivity to anti-VEGF treatment [39]. Thirdly, macrophages in surgically excised human CNV membranes following bevacizumab treatment have increased density and proliferative activity [78], and the proportion of circulating CD11b+ monocytes correlates with the number of anti-VEGF injections in patients with nAMD and PCV [79]. The actions of lipid-laden macrophages are also consistent with the well-established roles of monocytes and macrophages in promoting arteriogenesis by releasing growth factors, proteases and chemokines that mediate structural remodeling of the extracellular matrices, cell proliferation, and migration [71,77,80,81]. Both preclinical and clinical studies are consistent with the involvement of neovascular remodeling, in which macrophages are known to play important roles in anti-VEGF resistance [9,14,80].

Consistent with the contributions of lipid-laden macrophages in human arteriolar CNV formation, McLeod et al. identified a high frequency of activated HLA-DR^+^ macrophages associated with arteriolar CNV in human postmortem CNV specimens (Figures 9 and 10 in Ref. [82]). In addition to lipid-containing microglial cells found in type 3 neovascularization [83], hyperreflective lipid-filled cells of monocyte origin (i.e., macrophages) have been detected in nAMD [84]. Curcio and colleagues suggest that these monocyte-derived cells filled with lipid droplets resemble foam cells in coronary artery plaques [84], which are well-known to promote inflammation in association with atherosclerosis. Oxidized lipoproteins and macrophages were colocalized with CNV lesions, and most macrophages in the CNV membranes expressed oxidized lipoprotein-specific scavenger receptors, suggesting a close link between oxidized lipoproteins and macrophages in AMD [85]. Transcriptomic profiling showed that impaired cholesterol homeostasis is perturbed in aged macrophages and that oxysterol signatures in patient samples distinguish AMD from physiologic aging [86]. Expression of ABCA1 and cholesterol efflux are reduced in aged macrophages in mice and humans (old people and AMD) [87], and ABCA1 polymorphisms are associated with advanced AMD [88]. Multiple studies confirm the involvement of dysregulated lipid metabolism, macrophages, and inflammation in CNV [61,78,86,87,89,90,91,92,93,94,95,96,97,98,99,100], as well as the beneficial roles of lipid-lowering medications in reducing the risk of CNV, diabetic retinopathy, and diabetic macular edema [101,102,103,104]. It should be stressed that it can be difficult to definitively distinguish between microglia and macrophages by in vivo imaging of human patients. Although our macrophage depletion experiments suggest that blood-derived macrophages contribute to anti-VEGF resistance [39], retinal microglia may also be involved in anti-VEGF resistance.

Positive and negative roles have been assigned to macrophages in the progression of CNV pathogenesis. Macrophages may play a beneficial role in eliminating drusen and waste products, potentially reducing the formation of CNV [105,106]. Macrophages from young but not old mice inhibit experimental CNV [87,93]. Nevertheless, substantial evidence using multiple criteria, including histology and genetics, in both animal models and human patients supports the involvement of macrophages in CNV pathogenesis, particularly evident in their consistent presence within CNV lesions expressing elevated VEGF [61,79,87,89,90,93,94,95,96,97,98,107]. Macrophage activation is also associated with CNV [107]. Notably, during subretinal neovascularization, endothelial cells migrate through defects in Bruch’s membrane, which is primarily composed of elastin and collagen. Macrophages, expressing MMPs, contribute to the breakdown of Bruch’s membrane. Our data suggest that cholesterol dysregulation, inflammation, and macrophage activation underline the pathological role of aged macrophages in anti-VEGF resistance. Thus, we propose a strategic approach to manage anti-VEGF resistance by selectively targeting activated inflammatory macrophages. This can be achieved by normalizing the lipid rafts of activated macrophages, referred to as “inflammarafts [108,109]”, in CNV lesions (see “Section 5” below). This approach ensures the targeted intervention of the pathology without compromising the protective functions of macrophages at various stages of lesion progression.

## 5. Treatment Strategies for Anti-VEGF Resistance by Simultaneously Targeting Capillary and Arteriolar CNV

Our results suggest that while VEGF-dependent capillary angiogenesis is dominant in the CNV pathogenesis of young mice, inflammation-dependent neovascular remodeling and arteriolar CNV formation involving macrophages become dominant in aged mice and contribute to anti-VEGF resistance. Therefore, an effective treatment strategy requires the targeting of both capillary and arteriolar CNV. Because CNV is driven by abnormal levels of angiogenesis and inflammation with critical roles for VEGF-A, endothelial cells, and macrophages, we explored a new treatment strategy that targets each of these central elements to address the limitations of current anti-VEGF [39,40].

**Figure 3:**
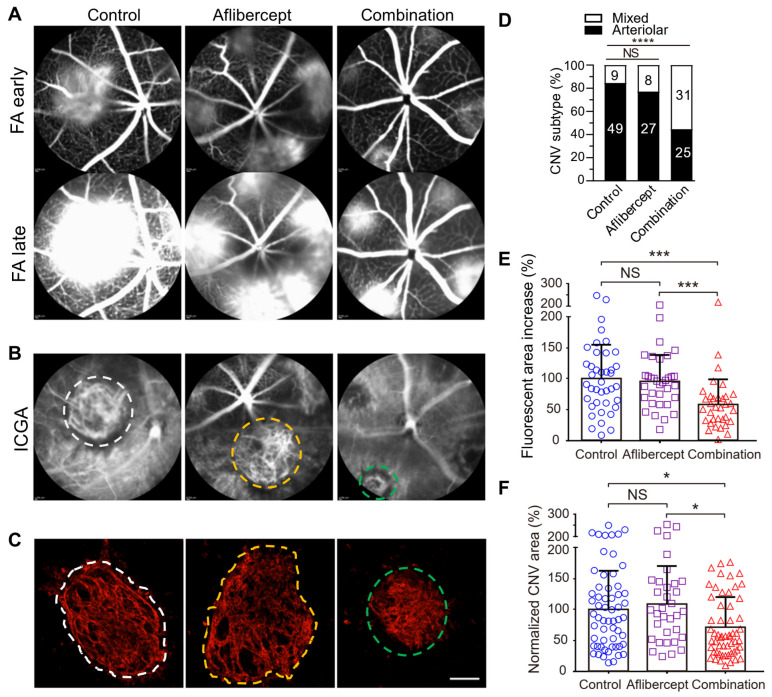
Comparison between aflibercept and combination therapy (AIBP, apoA-I, and aflibercept) in suppressing laser-induced CNV in old mice. Representative (**A**) FA, (**B**) ICGA, and (**C**) Alexa 568 isolectin labeled RPE/choroid flatmounts of CNV lesions after treatments. (**D**) CNV vessel type quantification based on isolectin-B4 staining. The numbers inside the bars indicate the number of CNV laser spots. (**E**) Quantitative results of the percentage increase of fluorescent area in CNV lesions between the early and late phases of FA. (**F**) Quantitative results of normalized CNV area. Old mice were treated on day 2 (**A**–**E**) and were analyzed at day 7 post laser injury. White and yellow dashed circles indicate arteriolar CNV in control and aflibercept treated mice. Green dashed circles indicate mixed type CNV in combination therapy treated mice in B and C. Mice treated on day 4 showed similar results. Bars represent mean ± SD. NS, *p* > 0.05; *, *p* < 0.05; ***, *p* < 0.001; ****, *p* < 0.0001. Adapted from Ref. [40].

Cholesterol-rich lipid rafts harboring activated receptors (e.g., VEGFR2, TLR4) serve as the organizing platform to initiate angiogenic and inflammatory signaling [108,110,111,112]. Extracellular apolipoprotein A-I (apoA-I) binding protein (AIBP) regulates lipid rafts via augmenting cholesterol efflux from endothelial cells, macrophages, and T cells, resulting in inhibition of angiogenesis and atherosclerosis, etc. [39,113,114,115,116,117,118,119,120]. AIBP binds its partner apoA-I or high-density lipoprotein (HDL), to enhance cholesterol efflux and inhibit lipid raft-anchored VEGFR2 signaling in endothelial cells [39,113]. By binding to the Toll-like receptor 4 (TLR4), AIBP/apoA-I augments cholesterol efflux from macrophages and microglia, normalizes plasma lipid rafts, and suppresses inflammation [109,114,115,121]. The ability of AIBP to target both hyperactive endothelial cells and cholesterol-laden macrophages makes it an ideal candidate to address the challenge of anti-VEGF resistance in CNV treatment. We found that a combination of AIBP/apoA-I and anti-VEGF treatment ameliorated anti-VEGF resistance to aflibercept in experimental CNV in old mice by robustly inhibiting arteriolar CNV (Figure 3) [40]. Despite sharing endothelial VEGFR2 signaling as a common target, combined AIBP and anti-VEGF provide synergistic therapeutic benefits for CNV. This is because macrophages that are recruited by VEGF to lesion sites of inflammation secrete additional VEGF and other pro-angiogenic factors, thereby creating strong positive feedback loops [91,92,122]. Thus, both anti-VEGF agents and AIBP are required to interrupt the vicious cycle of events initiated by the reciprocal causal nexus of VEGF and inflammation.

As discussed above, macrophages may have varying roles in CNV. How can we ensure the proposed combination therapy only targets pathological macrophages? This is achieved through the selectivity and normalization properties of AIBP on the lipid rafts of activated target cells. Previous studies have shown that AIBP selectively targets lipid rafts of activated macrophages/microglia and inhibits inflammatory signaling by binding to activated (e.g., dimerized) TLR4 [108,109,121]. AIBP normalizes lipid rafts of activated macrophages/microglia (i.e., inflammarafts [108]) [109], reducing the proinflammatory and proangiogenic subtypes (i.e., pathogenic) without affecting normal macrophage function, including their protective functions.

Out of the three components, infusion of HDL/apoA-I had been tested in clinical trials in the treatment of atherosclerosis. Whereas HDL/apoA-1 targeted therapies successfully ameliorate plaque in atherosclerosis mouse models, clinical trials failed to show a significant reduction in human atheroma (reviewed in Ref. [123]). Multiple possible reasons may account for the different responses of humans versus animal models to HDL/apoA-1 replacement therapy. One study reported that raising apoA-1 had striking stage-specific atheroprotective effects [124]. When initiated at early stages of disease, apoA-I markedly inhibited atheroma progression and systemic inflammation, but these benefits were attenuated when treatment was initiated at later times in mice with advanced atheroma. Most preclinical studies reporting such benefits were performed in young mice with early stage lesions [125,126,127], whereas large-scale HDL-raising clinical trials in elderly patients with established plaque failed to show benefit. This is antiparallel to our studies that demonstrate the efficacy of combination AIBP/apoA-I/anti-VEGF therapy in old mice with severe arteriolar CNV lesions that resemble arteriolar CNV in anti-VEGF-resistant AMD patients [40]. In addition, we have shown that: (1) apoA-I alone is insufficient to treat laser-induced CNV (Figure 6a,b in Ref. [39]); and (2) AIBP/apoA-I is insufficient to treat arteriolar CNV in old mice (Figure 6e in Ref. [39]). The likely reason is that AIBP can significantly enhance apoA-I’s ability to remove cholesterol from target cells (e.g., macrophages and endothelial cells) [39,109,113,114,115,121]. That is why we propose to develop the AIBP/apoA-I/anti-VEGF combination therapy.

## 6. How Does the Combination Therapy Compare with Anti-VEGF Gene Therapy and Higher Dose Anti-VEGF Regimen Currently in Development?

**Figure 4:**
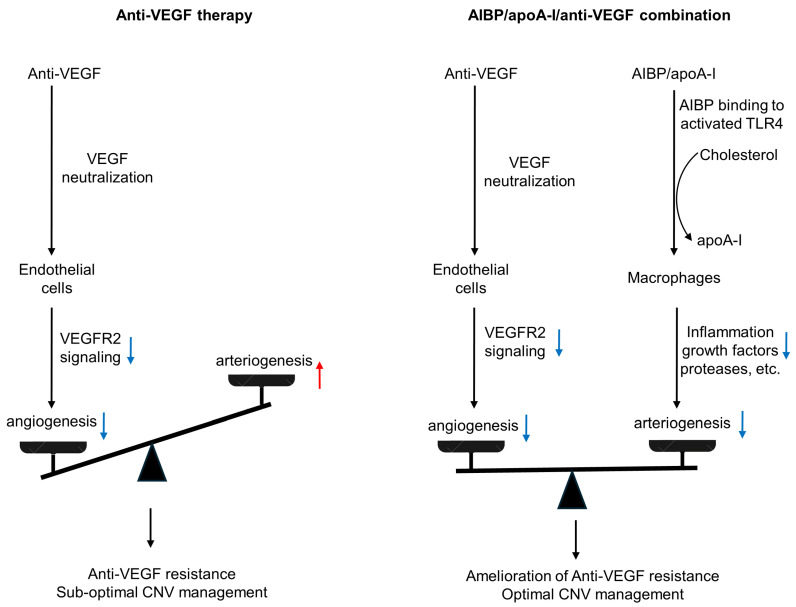
Comparison of anti-VEGF monotherapy with AIBP/apoA-I/anti-VEGF combination therapy in the treatment of CNV. Anti-VEGF therapies neutralize VEGF, inhibit VEGFR2 signaling in endothelial cells, and thereby inhibit angiogenesis and capillary CNV. However, this treatment results in unchecked arteriogenesis, vessel abnormalization, and arteriolar CNV formation, leading to anti-VEGF resistance and sub-optimal CNV management. In AIBP/apoA-I/anti-VEGF combination therapy, AIBP binds to activated TLR4 and augments cholesterol efflux from macrophages and microglia to apoA-I, normalizing plasma lipid rafts and suppressing inflammation, which inhibits arteriolar CNV. Simultaneously, anti-VEGF therapies inhibit VEGFR2 signaling in endothelial cells, thereby suppressing angiogenesis and capillary CNV. Thus, the combination therapy leads to the amelioration of anti-VEGF resistance and optimal CNV management. Blue arrows indicate signaling inhibition. The red arrow indicates signaling activation.

AMD is a complex, multi-factorial disease. It is unrealistic to expect that targeting one factor or one pathway will solve all the problems. The anti-VEGF gene therapy and higher dose regimen that are currently in development target VEGF-dependent angiogenesis without targeting arteriogenesis, which are unlikely to resolve resistance (see Discussion regarding high dose regimen in Section 1). In the HARBOR trial, high-dose ranibizumab (2.0 mg) did not increase efficacy in treatment-naïve patients [128]. In the recently completed PULSAR trial, 8 mg aflibercept sustained improvements in visual acuity and retinal anatomy at 22 months with 36% fewer injections relative to the standard 2-mg dose, suggesting the potential to reduce treatment burdens. However, there is no evidence the high-dose aflibercept eliminates anti-VEGF resistance. Rather, there is evidence that unbalanced treatments targeting VEGF-dependent angiogenesis alone can cause vessel abnormalization, arteriolar CNV formation, and anti-VEGF resistance [14,38] (Figure 4). Combination therapy has an advantage by targeting both angiogenesis and arteriogenesis.

## 7. Perspectives

Because the long-term efficacy of anti-VEGF therapy is suboptimal and repeated anti-VEGF treatment can lead to arteriolar CNV and anti-VEGF resistance [14,38], we predict that combination therapy with AIBP/apoA-I/anti-VEGF not only overcomes anti-VEGF resistance for monotherapy non-responders but also improves therapeutic efficacy at all levels of anti-VEGF response in the treatment of nAMD. To our knowledge, there is no treatment available for arteriolar CNV. Combination therapy has the potential to replace current anti-VEGF monotherapies and become a new first-line therapy. The global anti-VEGF therapeutics market was valued at USD 12.3 billion in 2022 and is estimated to reach USD 13.7 billion by 2031, representing a significant portion of global healthcare costs. Our objective is to generate preclinical efficacy and safety data to support an Investigational New Drug (IND) application for AIBP/apoA-I/aflibercept therapy and advance to a first-in-human Phase I clinical trial that will ultimately benefit a wide range of nAMD patients, including anti-VEGF non-responders and responders with sub-optimal long-term efficacy.
